# Supplementary material for: SynDRep: a synergistic partner prediction tool based on knowledge graph for drug repurposing
Source: Bioinform Adv. 2025 Jun 5;5(1):vbaf092. doi: 10.1093/bioadv/vbaf092 (PMC12148216; doi:10.1093/bioadv/vbaf092)
Supplement: vbaf092_Supplementary_Data [file vbaf092_supplementary_data.docx]

SynDRep: A Synergistic Partner Prediction Tool based on Knowledge Graph for Drug Repurposing

**Karim S. Shalaby^1,2^, Sathvik Guru Rao^1^, Bruce Schultz^3^, Martin Hofmann-Apitius^1,4^, Alpha Tom Kodamullil^1,5,*,+^, Vinay Srinivas Bharadhwaj^1,4,+^**

1. Department of Bioinformatics, Fraunhofer Institute for Algorithms and Scientific Computing, 53757 Sankt Augustin, Germany
2. Department of Pharmaceutics and Industrial Pharmacy, Faculty of Pharmacy, Ain Shams University, 11566 Cairo, Egypt
3. Institute of Biomedical Informatics, University Hospital Cologne, 50937 Cologne, Germany
4. Bonn-Aachen International Center for Information Technology (B-IT), University of Bonn, 53115 Bonn, Germany
5. Causality Biomodels, Kinfra Hi-Tech Park, Kalamassery, Cochin, 683503 Kerala, India

***Corresponding Author**: Alpha Tom Kodamullil, Department of Bioinformatics, Fraunhofer Institute for Algorithms and Scientific Computing (SCAI), Sankt Augustin 53757, Germany. Telephone details: +49 2241 14-4050. Email: [alpha.tom.kodamullil@scai.fraunhofer.de](mailto:alpha.tom.kodamullil@scai.fraunhofer.de)

**^+^** These authors contributed equally to this work.

Supplementary

# Results

## Classical Machine learning

A nested cross-validation was used to assess the performance of the five machine learning (ML) models.  Since support vector machine models didn’t converge within the time limit for HPO, only four of the five ML models were assessed according to their resulted average ROC-AUC score. As depicted in Figure S4, there is a negligible difference between the ROC-AUC scores of four ML models. However, the elastic net model exhibited the highest ROC-AUC (0.8027) and was selected for the further prediction of synergism.

Synergy predictions between pairs of drugs in the pharmacome were carried out using the trained elastic net model. The predictions were divided based on the predicted relation between drugs into two distinct subsets: a synergy set, and an antagonism set. This categorization yielded a synergism set comprising 3,330,649 combinations and an antagonism set comprising 7,764,165 combinations. Our subsequent focus centered on the synergy set to validate the model’s predictability further. The five highest-scoring synergy combinations, in terms of synergism class probability, comprise aprinocarsen with carboxymycobactin T, salmon calcitonin, vintafolide, teniposide, or bevasiranib. They were subjected to a thorough literature review to virtually validate the reliability of the model's predictions. Although most of These drugs are used for similar indications as anti-cancers, we could not find any supporting studies validating their synergy when used in combination.

## KG embedding

The produced hyperparameters from the hyperparameter optimization (HPO) (Supplementary Table S1) were used to train the optimum model, which was then evaluated by the percentage of true predictions for all relations. RotatE was the best model to produce true predictions at the lowest rank (73.09%) as shown in Figure S5. We characterized this model results further by calculating the different models’ multi-class ROC-AUC. RotatE also produced the highest multiclass ROC-AUC (0.76), which indicated the model’s high performance in the prediction of the proper relation compared to other models (Figure S6). An additional test set exclusively containing drug-drug relations from the original test set was employed to validate RotatE as the optimal model for link prediction among drug entities within the pharmacome. The results demonstrated that RotatE consistently outperformed other models, exhibiting the highest percentage of accurate predictions at the lowest rank within this dedicated subset (61.84%), as illustrated in Figure 2.

## Synergy prediction

The set of predictions was divided based on the predicted relation between the head and tail entities, creating two distinct subsets: a synergy set (52,127 combinations) and an antagonism set (35,684 combinations). Our subsequent focus centered on the synergy set to assess the model’s predictability further and select potential drug repurposing candidates. The five highest-scoring synergy combinations are shown in supplementary Table S2.

## Drug repurposing

Based on our predictions, we selected a list of drugs that exhibited the highest score as synergistic partners with selected commonly prescribed drugs for the disease of interest. Then we chose the safe drugs and explored their pathways to the disease in the pharmacome.

### Alzheimer’s candidates

To find repurposing candidates for Alzheimer’s disease (AD), we detected the safe predicted synergistic partners with donepezil, rivastigmine, and galantamine. Mefloquine is an orally administered blood antimalarial (Palmer, Holliday and Brogden 1993) and was reported in our predicted synergy with both donepezil and rivastigmine. Moreover, Studies have shown that mefloquine significantly enhances the procognitive effect of donepezil in C57BL/6 male mice and Sprague–Dawley (8-week-old) male rats, and potentiates the donepezil-induced hemodynamic effects in the hippocampus of C57BL/6 male mice (Droguerre *et al.* 2020; Vidal *et al.* 2020). Our common pathway exploration from pharmacome revealed a pathway within our pharmacome between mefloquine and both donepezil and rivastigmine (Figure S7). Other AD repurposing candidates are ciprofloxacin, moxifloxacin, chloroquine, taribavirin, and ivermectin. They appeared in synergistic combinations with one or more drugs prescribed for AD. The common pathways in pharmacome were elucidated and some of them are shown in Supplementary Figure S8 and Figure S9, and many studies, in cell and animal models as well as clinical trials,  support their potential use for the management of AD (Zbarsky, Thomas and Greenfield 2004; Osorio *et al.* 2019; Zusso *et al.* 2019; Lindblom *et al.* 2021; Niklasson, Klitz and Lindquist 2022; Varma *et al.* 2023). Atovaquone has shown unclear pathways in our KG and no supporting published studies for its use in AD, therefore we suggest it is the least suitable for repurposing.

### Schizophrenia and bipolar disorder candidates

Mefloquine stemmed from our synergy prediction with the three selected drugs for the management of Schizophrenia: olanzapine, ziprasidone, and thiothixene, although the majority of published animal models and retrospective studies demonstrate that its prolonged use induced psychosis and might worsen Schizophrenia (Alisky, Chertkova and Iczkowski 2006; Mawson 2013). The shared pathways to schizophrenia between mefloquine and the three drugs are very similar (Figure S10). Like mefloquine, the majority of published studies demonstrate that extended use of chloroquine induced psychosis and might worsen Schizophrenia (Alisky, Chertkova and Iczkowski 2006; Biswas, Sen and Majumdar 2014). However, it stems from our synergy prediction with ziprasidone and thiothixene (Supplementary Figure S11). On the other hand, pyrimethamine, an antiparasitic drug typically used to treat malaria and toxoplasmosis (Wishart *et al.* 2018), has no existing studies investigating the pure potential effect of pyrimethamine on Schizophrenia. However, most of them are discussing it in the context of reducing the symptoms of toxoplasmosis-induced schizophrenia (Webster *et al.* 2006; Castaño *et al.* 2022). The presence of pyrimethamine as a synergistic drug with ziprasidone and thiothixene highlights the need for further research on the underlying mechanisms and pathways for its use in the control of schizophrenia, even in the absence of Toxoplasmosis.

## Causal-only pharmacome

miconazole and albendazole have emerged as promising candidates for repurposing to treat AD. A prominent pathway of miconazole in association with donepezil and rivastigmine, and galantamine to AD involves the role of these three drugs as inhibitors of acetylcholinesterase (ACHE). ACHE leads to an increase in amyloid-beta precursor protein (APP) through increasing the abundance of beta-amyloid. This elevation in APP has been linked to an increased AD. Miconazole, on the other hand, acts as an inhibitor to nitric oxide synthase 2 (NOS2). NOS2 also leads to an increase in APP through increasing the abundance of nitric oxide. Since all drugs are inhibitors, they inhibit these pathways resulting in protection from AD or slowing its progression.

Albendazole shares a shared pathway to AD with donepezil and rivastigmine. As previously mentioned, the donepezil and rivastigmine pathway involves the indirect inhibition of the abundance of beta-amyloid.  In addition to increasing APP, beta-amyloid typically increases phosphorylated MAPT which is a hallmark of AD progression. Therefore, the reduction in beta-amyloid leads to inhibition of phosphorylated MAPT. On the other hand, Albendazole inhibits Tubulin alpha-1A protein (TUBA1A), leading to a decrease in Cell division control protein 42 homolog (CDC42). The decrease in CDC42 will result in a decrease in a composite of mitogen-activated protein kinase 1 and 3 (MAPK1 and MAPK3), which will eventually decrease the phosphorylated MAPT.

# Hardware

KG embeddings were trained on a GPU node equipped with two Intel Xeon Scalable Gold 6140 processors, each featuring 18 cores and 36 threads (totaling 36 cores and 72 threads per node) operating at a base frequency of 2.3GHz and a turbo frequency of 3.7GHz. The node had 768GB of DDR4 ECC Reg RAM and was powered by eight NVIDIA Tesla V100 SXM2 GPUs, each with 32GB of RAM, totaling 5120 CUDA cores and 640 Tensor cores, along with 32GB of HBM2 memory and a 300GB/s NVIDIA NVLink interconnect. The networking was facilitated by a 100GBit/s Intel OmniPath, while storage comprised four 3.2TB Samsung NVMe SSD PM1725a drives for local intermediate data and utilized the BeeGFS parallel file system for home directories.

The training and evaluation of ML models took place on a symmetric multiprocessing (SMP) node featuring four Intel Xeon Platinum 8160 processors, each with 24 cores and 48 threads (totaling 96 cores and 192 threads), running at a base frequency of 2.1GHz and a turbo frequency of 3.7GHz. This node was equipped with 1536GB (1.5TB) of DDR4 ECC Reg RAM. Like the GPU node, it also utilized a 100GBit/s Intel OmniPath for networking, and its storage consisted of two Intel P4600 1.6TB U.2 PCIe NVMe drives for local intermediate data, with the BeeGFS parallel file system for managing home directories.

# References

Alisky JM, Chertkova EL, Iczkowski KA. Drug interactions and pharmacogenetic reactions are the basis for chloroquine and mefloquine-induced psychosis. *Med Hypotheses* 2006;**67**:1090–4.

Bista R, Lee DW, Pepper OB *et al.* Disulfiram overcomes bortezomib and cytarabine resistance in Down-syndrome-associated acute myeloid leukemia cells. *J Exp Clin Cancer Res* 2017;**36**:22.

Biswas PS, Sen D, Majumdar R. Psychosis following chloroquine ingestion: a 10-year comparative study from a malaria-hyperendemic district of India. *Gen Hosp Psychiatry* 2014;**36**:181–6.

Castaño BL, Silva AA, Hernandez-Velasco LL *et al.* Sulfadiazine Plus Pyrimethamine Therapy Reversed Multiple Behavioral and Neurocognitive Changes in Long-Term Chronic Toxoplasmosis by Reducing Brain Cyst Load and Inflammation-Related Alterations. *Front Immunol* 2022;**13**:822567.

Droguerre M, Duchêne A, Picoli C *et al.* Efficacy of THN201, a Combination of Donepezil and Mefloquine, to Reverse Neurocognitive Deficits in Alzheimer’s Disease. *Front Neurosci* 2020;**14**.

Fountzilas G, Inoue S, Ohnuma T. Schedule-dependent interaction of cytarabine plus doxorubicin or cytarabine plus mitoxantrone in acute myelocytic leukemia cells in culture. *Leukemia* 1990;**4**:321–4.

Kim SJ, Shin D-Y, Kim JS *et al.* A phase II study of everolimus (RAD001), an mTOR inhibitor plus CHOP for newly diagnosed peripheral T-cell lymphomas. *Ann Oncol* 2016;**27**:712–8.

Lindblom N, Lindquist L, Westman J *et al.* Potential Virus Involvement in Alzheimer’s Disease: Results from a Phase IIa Trial Evaluating Apovir, an Antiviral Drug Combination. *J Alzheimers Dis Rep* 2021;**5**:413–31.

Mawson AR. Mefloquine use, psychosis, and violence: A retinoid toxicity hypothesis. *Med Sci Monit Int Med J Exp Clin Res* 2013;**19**:579–83.

Niklasson B, Klitz W, Lindquist L. Positive Response of Alzheimer’s Disease Patients to Antiviral Therapy-Case Reports. *Ann Case Rep* 2022.

O’Reilly T, McSheehy PMJ, Wartmann M *et al.* Evaluation of the mTOR inhibitor, everolimus, in combination with cytotoxic antitumor agents using human tumor models in vitro and in vivo. *Anticancer Drugs* 2011;**22**:58–78.

Osorio C, Kanukuntla T, Diaz E *et al.* The Post-amyloid Era in Alzheimer’s Disease: Trust Your Gut Feeling. *Front Aging Neurosci* 2019;**11**.

Palmer KJ, Holliday SM, Brogden RN. Mefloquine. A review of its antimalarial activity, pharmacokinetic properties and therapeutic efficacy. *Drugs* 1993;**45**:430–75.

Partridge FA, Brown AE, Buckingham SD *et al.* An automated high-throughput system for phenotypic screening of chemical libraries on C. elegans and parasitic nematodes. *Int J Parasitol Drugs Drug Resist* 2017;**8**:8–21.

Varma VR, Desai RJ, Navakkode S *et al.* Hydroxychloroquine lowers Alzheimer’s disease and related dementias risk and rescues molecular phenotypes related to Alzheimer’s disease. *Mol Psychiatry* 2023;**28**:1312–26.

Vidal B, Droguerre M, Valdebenito M *et al.* Pharmaco-fUS for Characterizing Drugs for Alzheimer’s Disease – The Case of THN201, a Drug Combination of Donepezil Plus Mefloquine. *Front Neurosci* 2020;**14**:835.

Webster JP, Lamberton PHL, Donnelly CA *et al.* Parasites as causative agents of human affective disorders? The impact of anti-psychotic, mood-stabilizer and anti-parasite medication on Toxoplasma gondii’s ability to alter host behaviour. *Proc Biol Sci* 2006;**273**:1023–30.

Wishart DS, Feunang YD, Guo AC *et al.* DrugBank 5.0: a major update to the DrugBank database for 2018. *Nucleic Acids Res* 2018;**46**:D1074–82.

Yang W, Xie J, Hou R *et al.* Disulfiram/cytarabine eradicates a subset of acute myeloid leukemia stem cells with high aldehyde dehydrogenase expression. *Leuk Res* 2020;**92**:106351.

Yu Y-M, Bu F-Z, Meng S-S *et al.* The first marine dual-drug cocrystal of cytarabine with 5-fluorouracil having synergistic antitumor effects shows superior biopharmaceutical peculiarities by oral administration. *Int J Pharm* 2022;**629**:122386.

Zbarsky V, Thomas J, Greenfield S. Bioactivity of a peptide derived from acetylcholinesterase: involvement of an ivermectin-sensitive site on the alpha 7 nicotinic receptor. *Neurobiol Dis* 2004;**16**:283–9.

Zusso M, Lunardi V, Franceschini D *et al.* Ciprofloxacin and levofloxacin attenuate microglia inflammatory response via TLR4/NF-kB pathway. *J Neuroinflammation* 2019;**16**:148.

# Tables

**Table S1. The hyperparameter levels produced from the hyperparameter optimization process and used during the training of the optimum embedding models.**

| **Parameter** | **TransE** | **TransR** | **RotatE** | **ComplEx** | **HolE** |
| --- | --- | --- | --- | --- | --- |
| Embedding dimensions | 256 | 32 | 256 | 128 | 256 |
| Relation space dimensions | - | 224 | - | - | - |
| Scoring factor norm | L_1_ | L_1_ | - | - | - |
| Optimizer | Stochastic gradient descent | Stochastic gradient descent | Adam | AdaGrad | AdaGrad |
| Learning rate | 0.018 | 0.57 | 0.005 | 0.31 | 1 |
| Training batch size | 512 | 2048 | 256 | 1024 | 256 |
| Loss | Margin ranking loss | Margin ranking loss | Self-adversarial negative sampling loss | Softplus loss | Margin ranking loss |
| Self-adversarial sampling temperature | - | - | 0.7 | - | - |
| The margin between positive and negative scores | 3.5 | 0.5 | - | - | 1.5 |

**Table S2. The five top scorer predictions, based on the original HBP, and their validation from published studies.**

| **Rank** | **Drug A** | **Drug B** | **References** | **Remarks** | **Hit ratio** |
| --- | --- | --- | --- | --- | --- |
| 1 | Everolimus | Doxorubicin | (O’Reilly *et al.* 2011; Kim *et al.* 2016) | Studies showed an additive or a synergistic effect between the two drugs | 0.5 (2 supporting studies out of 4 retrieved studies) |
| 2 | Cytarabine | Fluorouracil | (Yu *et al.* 2022) | Cocrystal of cytarabine with 5-fluorouracil having synergistic antitumor effects | 0.33 (1 supporting studies out of 3 retrieved studies) |
| 3 | Mebendazole | Auranofin | - | There is no study for their combination | - |
| 4 | Disulfiram | Cytarabine | (Bista *et al.* 2017; Yang *et al.* 2020) | studies show the two drugs are routinely used in combination but no specific study on synergism | 1 (2 supporting studies out of 2 retrieved study) |
| 5 | Doxorubicin | Cytarabine | (Fountzilas, Inoue and Ohnuma 1990) | combination showed additive effect | 0.5 (1 supporting studies out of 2 retrieved study) |

# Figures


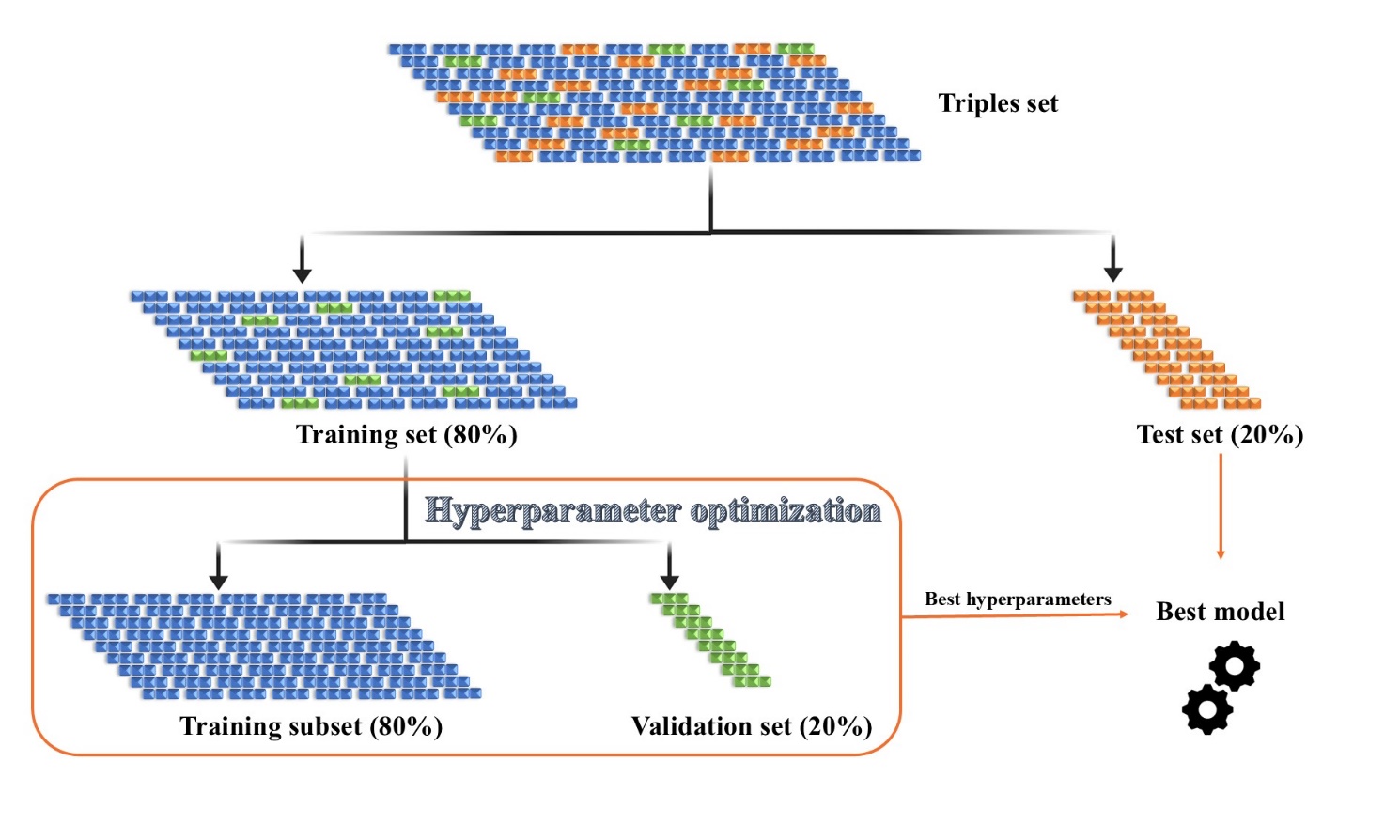


**Figure S1. The splitting of pharmacome triples for hyperparameter optimization and model training.** the set of triples (source-relation-target) that make up the network structure of pharmacome was then stratified using the PyKEEN into a training set (80%) and a test set (20%). To prevent the dissemination of the test set into the training set during the HPO or training of the model, we isolated the test set, and the training set was further split into training (80%) and validation (20%) sets. After obtaining the best hyperparameters, a model was trained, and its performance was evaluated using the test set.


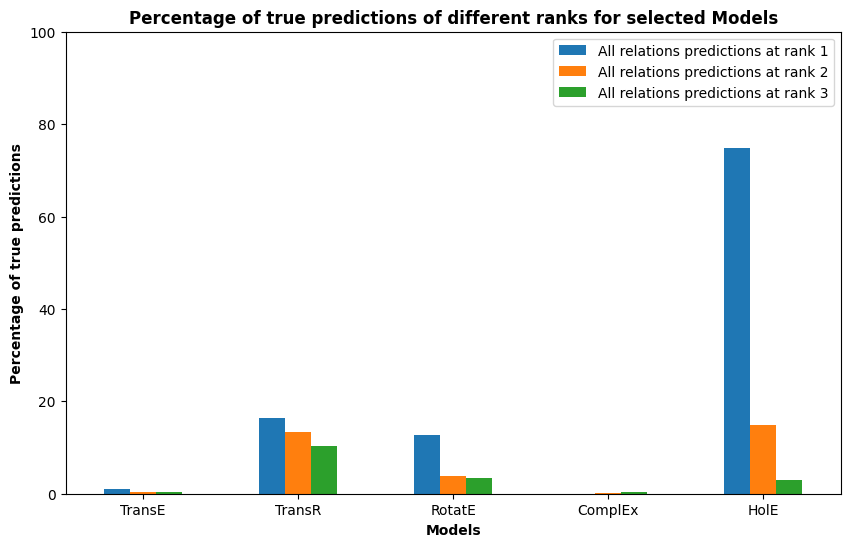


**Figure S2. Percentage of true all relations predictions at different ranks for selected models used to embed Casual-only pharmacome.** Optimum models were used to predict the test set, and then the predicted relations were compared to the actual relations to calculate the percentage of true predictions.


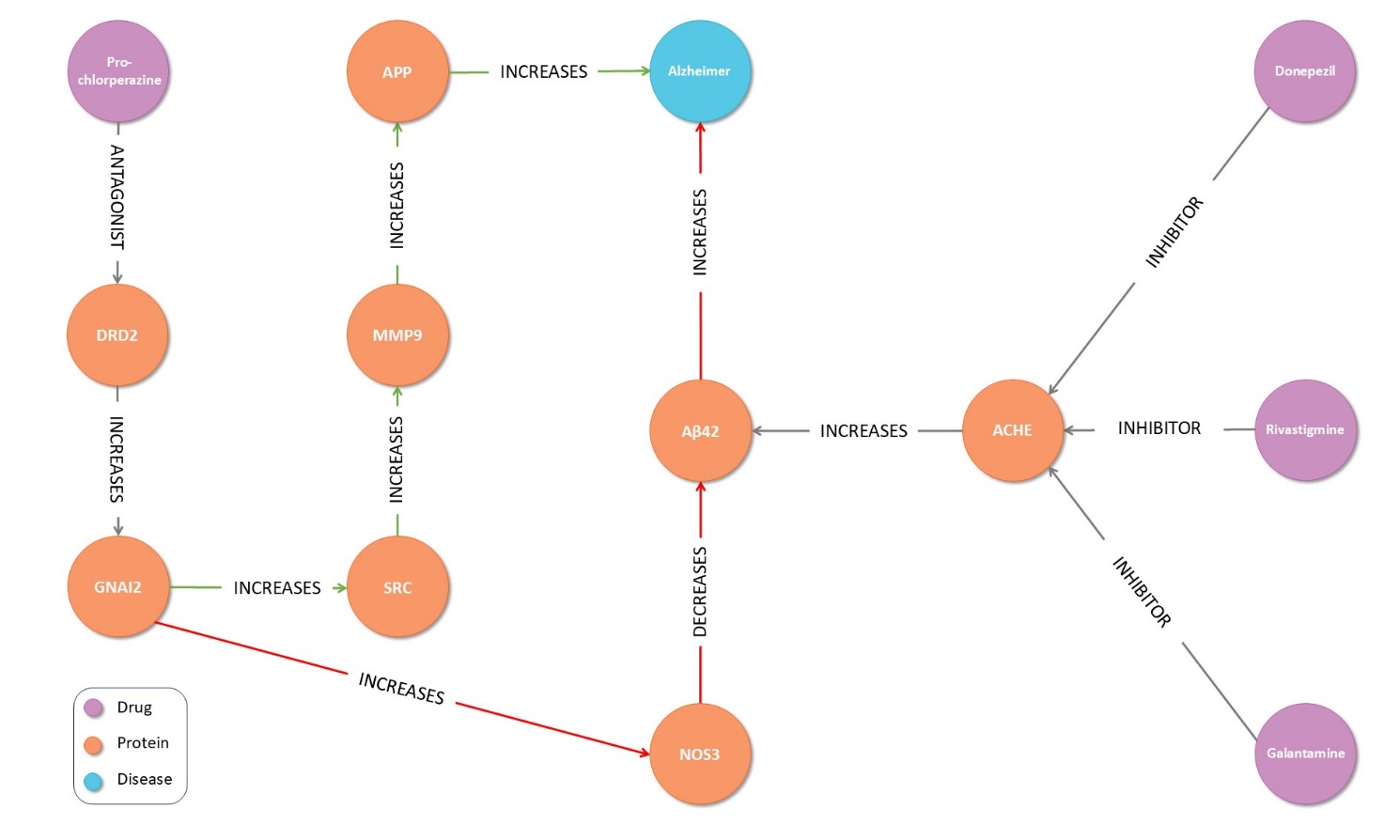


**Figure S3. The shared pathways between prochlorperazine, donepezil, rivastigmine, and galantamine to Alzheimer’s disease.** Prochlorperazine has two pathways that affect Alzheimer’s disease. The red pathway increases Alzheimer’s disease, while the green pathway decreases it.


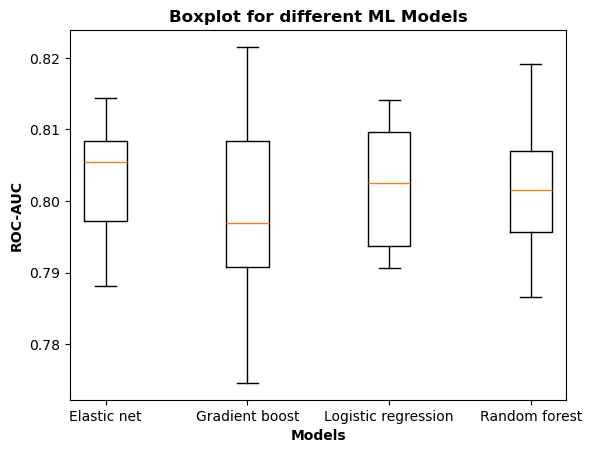


**Figure S4. Benchmarking of four machine learning models trained to classify between synergism and antagonism based on physicochemical properties of drugs and topological features from HBP.** Each boxplot shows the distribution of the ROC-AUC values over ten repeats of the ten-fold nested cross-validation procedure.


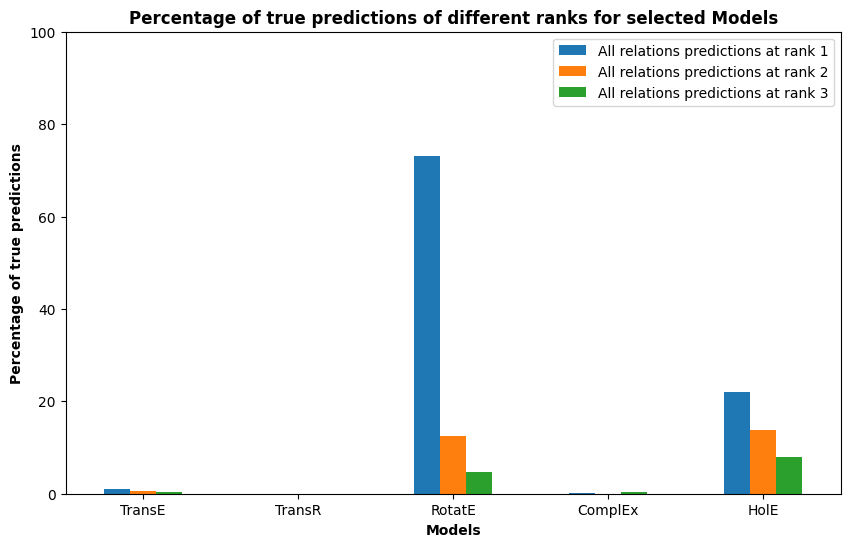


**Figure S5. Percentage of true predictions for all relations at different ranks for selected models.** Optimum models were used to predict the test set from the original HBP, and then the predicted relations were compared to the actual relations to calculate the percentage of true predictions.


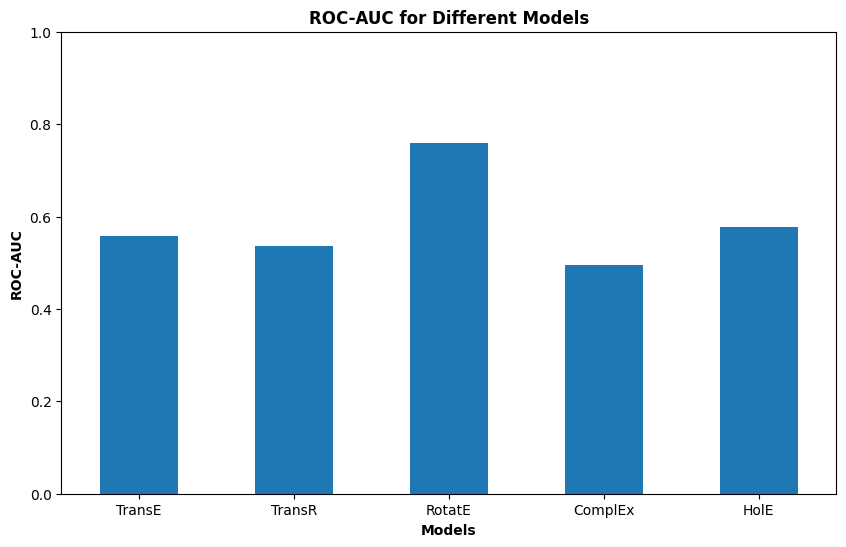


**Figure S6. Multi-class ROC-AUC for all relations predictions at rank 1 for selected model.**


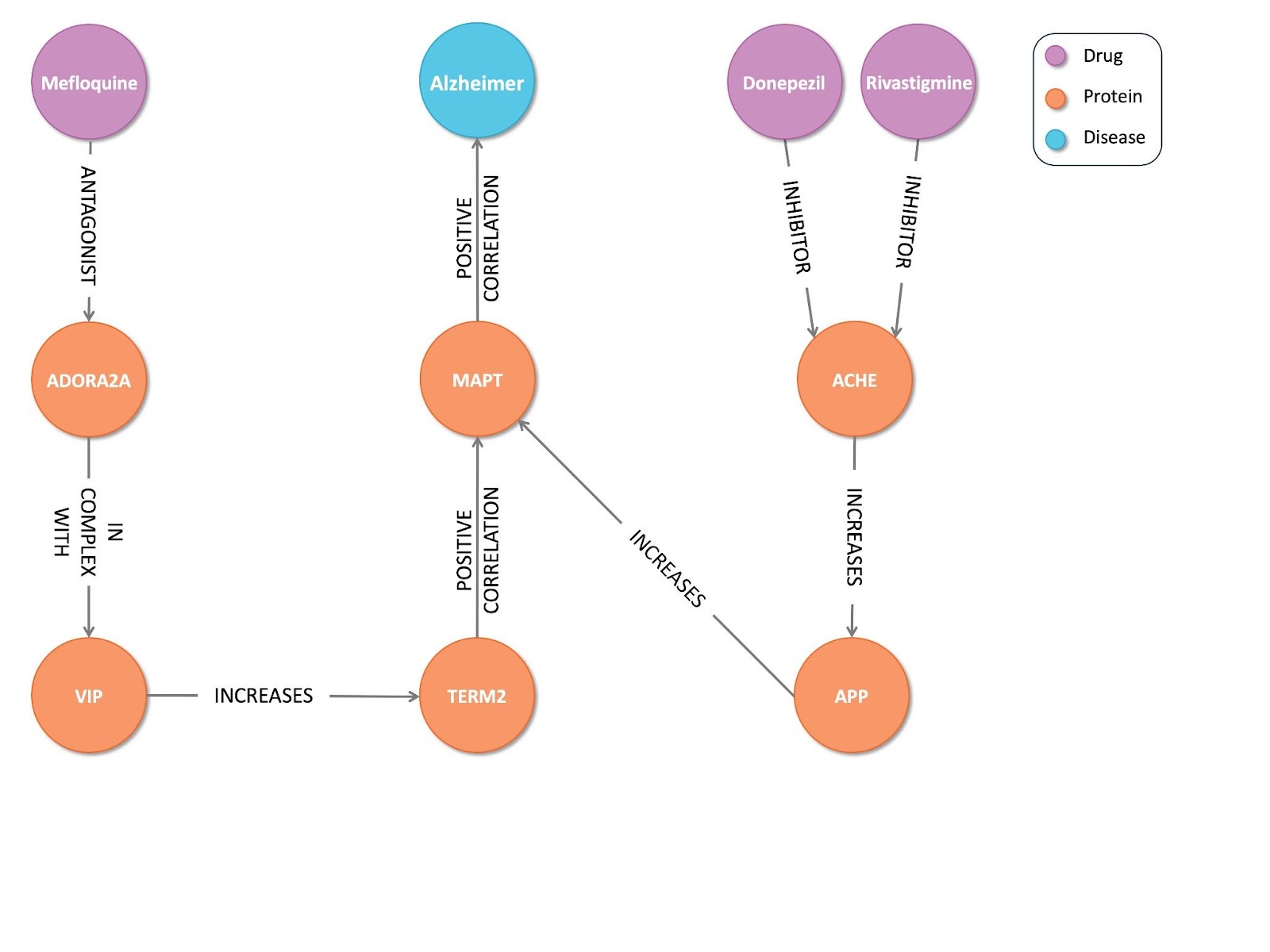


**Figure S7. The shared pathway between mefloquine and both donepezil and rivastigmine to Alzheimer’s disease.**


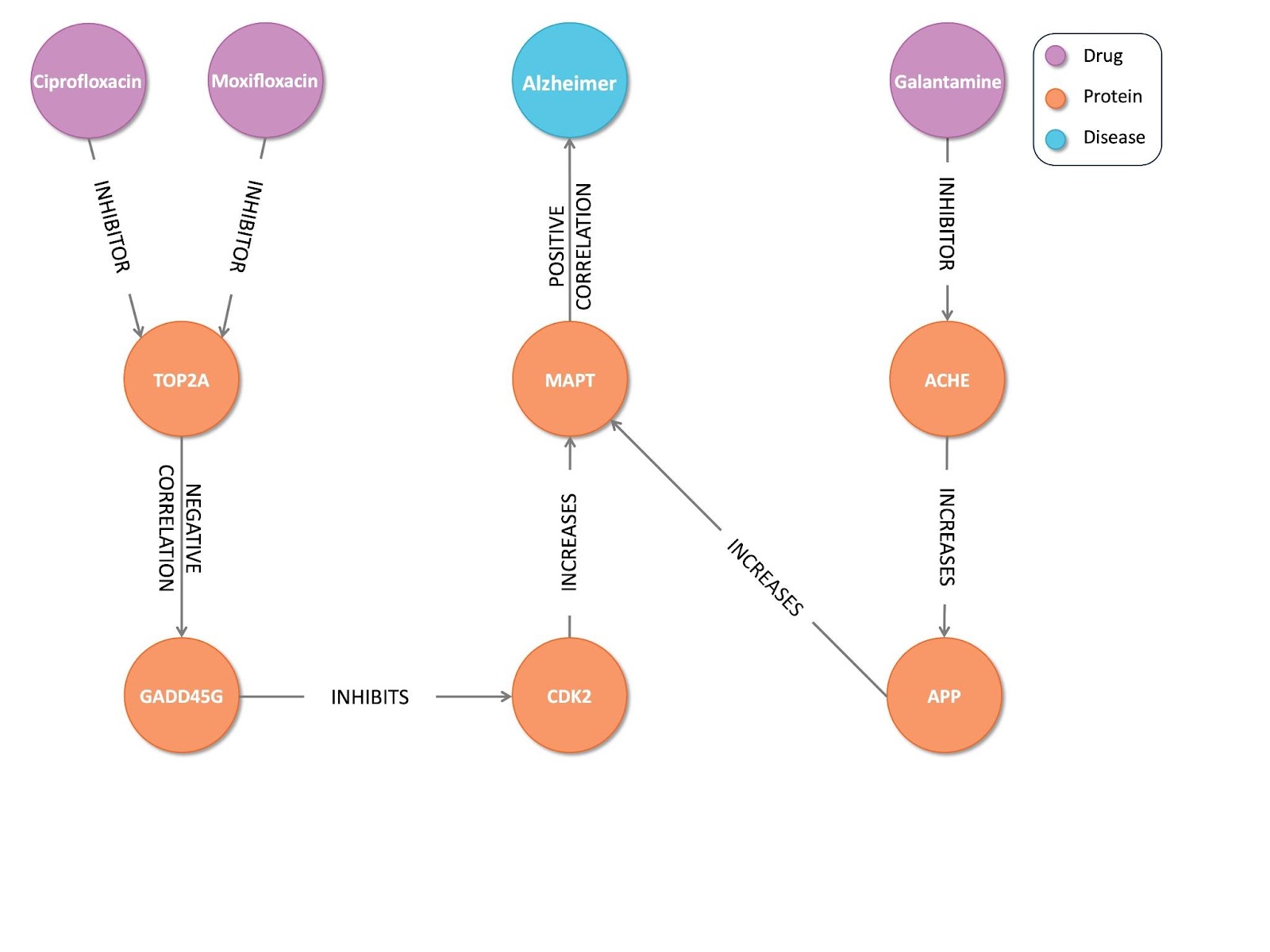


**Figure S8. The shared pathway between galantamine and both ciprofloxacin and moxifloxacin to Alzheimer’s disease.**


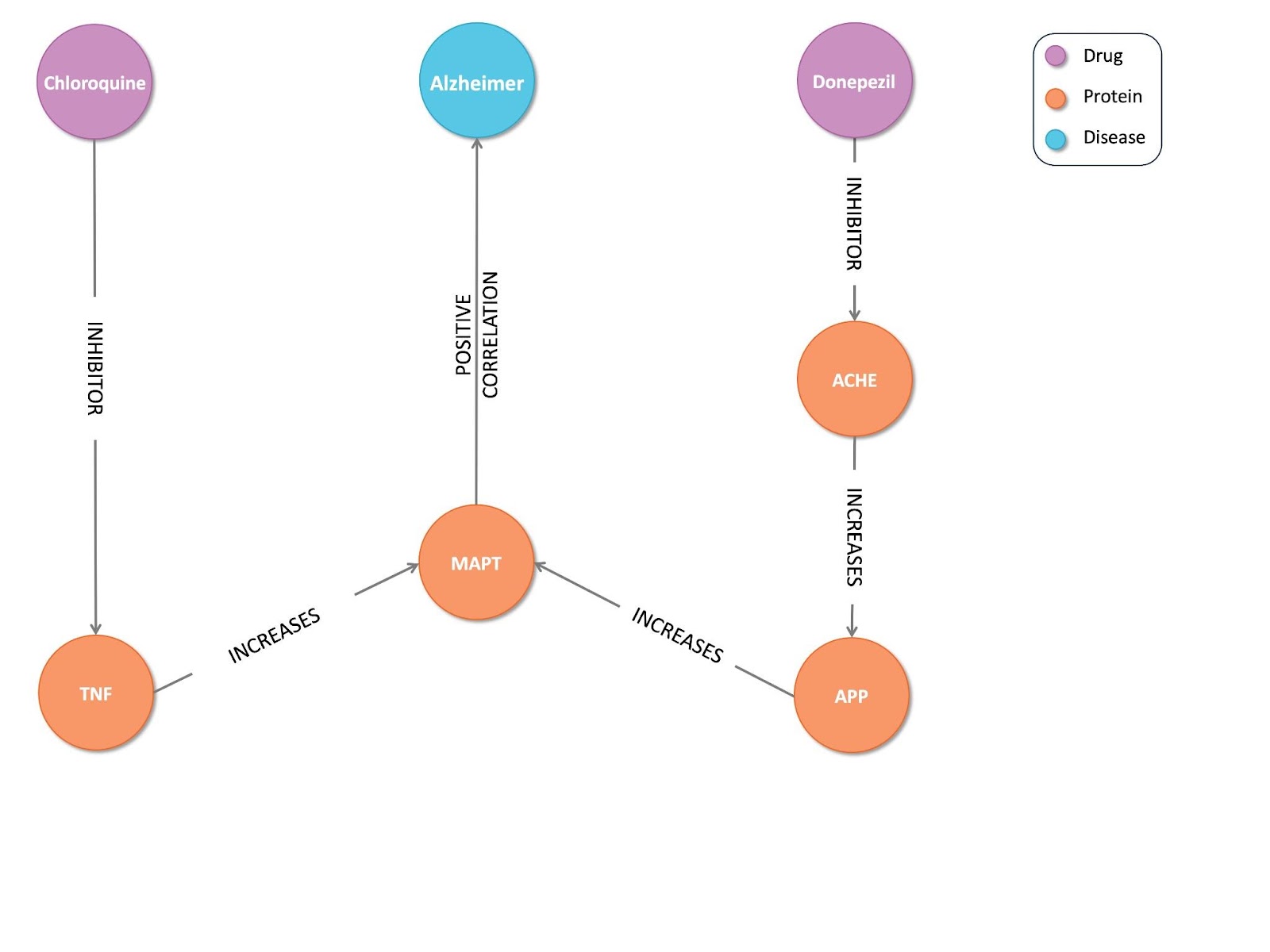


**Figure S9. The shared pathway between donepezil and chloroquine to Alzheimer’s disease.**


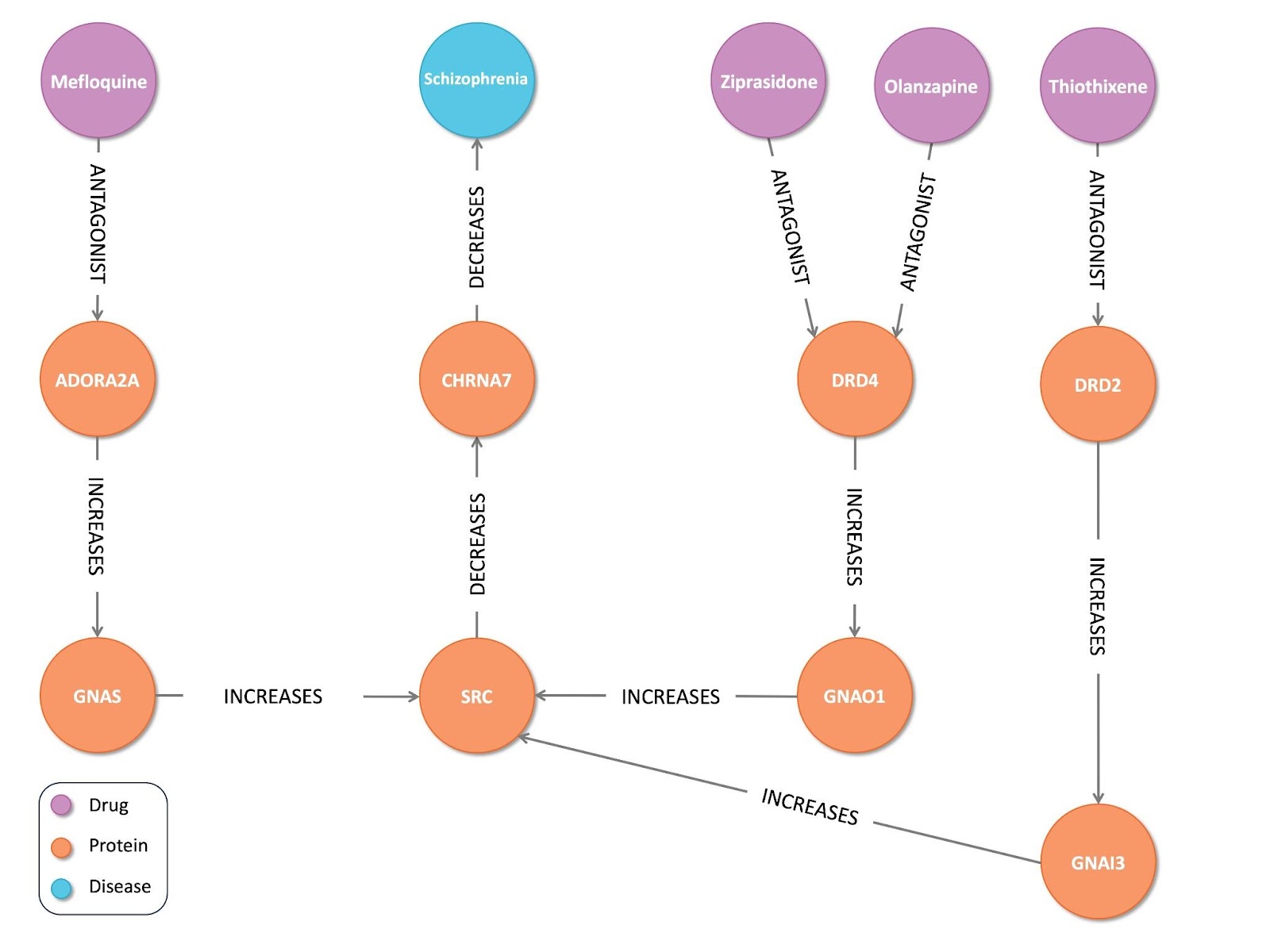


**Figure S10. The shared pathways between mefloquine, olanzapine, ziprasidone, and Thiothixene to Schizophrenia.**


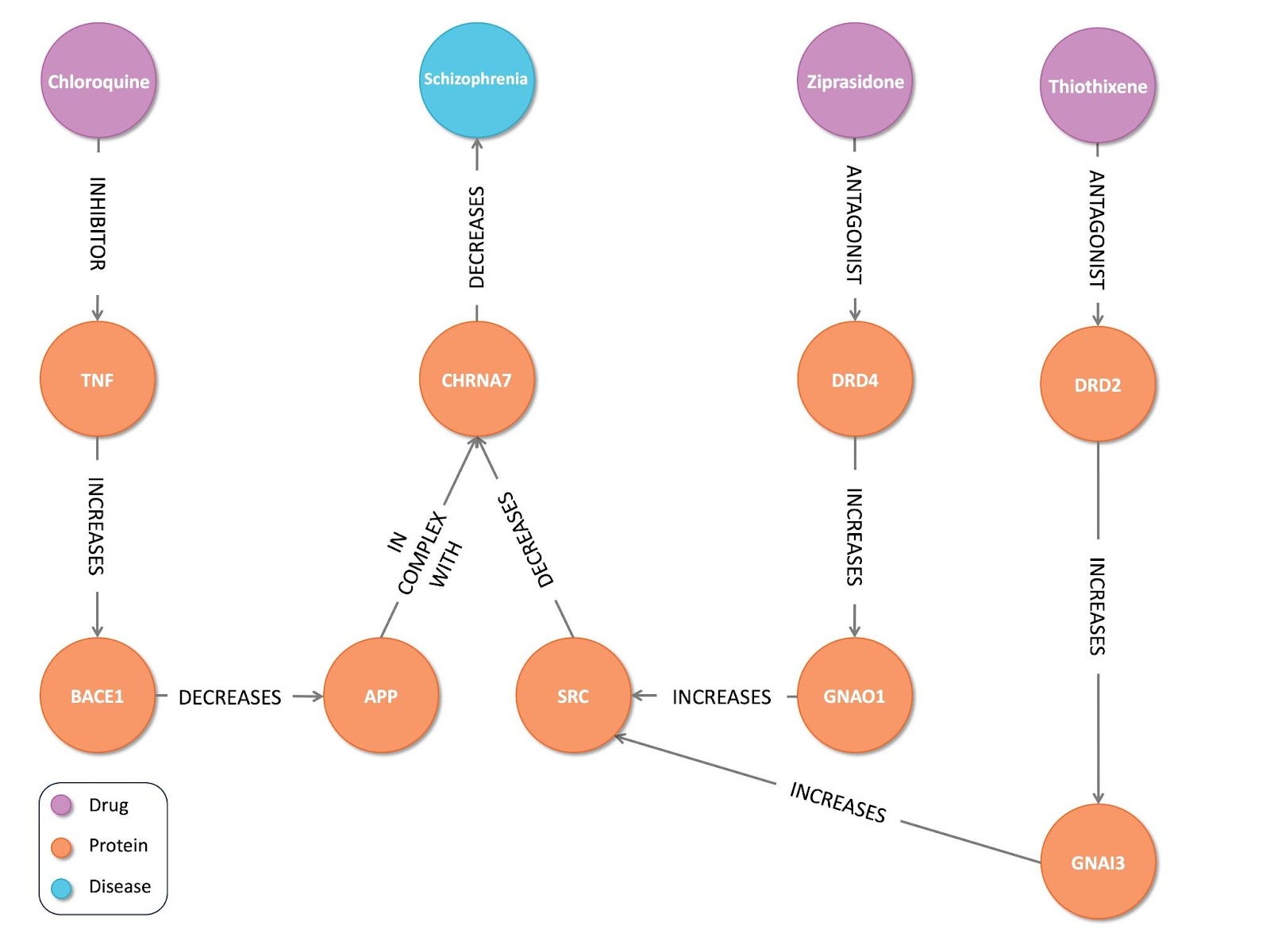


**Figure S11. The shared pathways between chloroquine, ziprasidone, and Thiothixene to Schizophrenia.**
